# Occurrence and Antimicrobial Susceptibility of Major Bacterial Pathogens Associated with Subclinical Mastitis in Dairy Cows in Western Romania

**DOI:** 10.3390/microorganisms14010026

**Published:** 2025-12-21

**Authors:** Răzvan-Dragoș Roșu, Adriana Morar, Emil Tîrziu, Viorel Herman, Alexandra Ban-Cucerzan, Sebastian Alexandru Popa, Răzvan-Tudor Pătrînjan, Alexandra Pocinoc, Bianca-Luisa Ghițan, Kálmán Imre

**Affiliations:** 1 Faculty of Veterinary Medicine, University of Life Sciences “King Mihai I” from Timişoara, 300645 Timișoara, Romania; razvan.rosu@usvt.ro (R.-D.R.); emiltirziu@usvt.ro (E.T.); viorel.herman@fmvt.ro (V.H.); alexandracucerzan@usvt.ro (A.B.-C.); sebastian.popa@usvt.ro (S.A.P.); razvan.patrinjan.fmv@usvt.ro (R.-T.P.); alexandra.pocinoc@usvt.ro (A.P.); bianca.ghitan.iosud@usvt.ro (B.-L.G.); 2 Research Institute for Biosecurity and Bioengineering, University of Life Sciences ‘’King Mihai I” from Timișoara, 300645 Timișoara, Romania

**Keywords:** bacterial pathogens, antimicrobial susceptibility, subclinical mastitis, dairy cows

## Abstract

Subclinical mastitis is a major but often overlooked constraint to dairy productivity, causing economic losses through reduced milk yield and quality. In Romania, comprehensive data on the bacterial etiology and antimicrobial resistance (AMR) of subclinical mastitis are limited. This study aimed to characterize the etiological agents and antimicrobial susceptibility profiles of major bacterial pathogens isolated from subclinical mastitis cases in dairy cows from Western Romania. Between 2021 and 2022, milk samples were collected from 117 lactating cows diagnosed with subclinical mastitis on three dairy farms. Bacterial isolation and differentiation were performed on ChromID^®^ CPS^®^ Elite Agar, and isolates were confirmed by standard biochemical tests. Antimicrobial susceptibility testing of *Staphylococcus aureus* and *Escherichia coli* isolates was conducted using the automated Vitek^®^2 system, interpreted according to CLSI veterinary standards. Multidrug resistance (MDR) was defined as resistance to at least one agent in three or more antimicrobial classes. Bacterial growth occurred in 51 of 117 samples (43.6%). *S. aureus* subsp. *aureus* predominated (28.2%), followed by *E. coli* (4.3%), *Klebsiella pneumoniae* subsp. *pneumoniae* (2.3%), and *Streptococcus uberis* (2.3%). Mixed infections occurred in 6.0% of positive samples. Among *S. aureus*, the highest resistance rates were to fosfomycin (58.3%), penicillin (44.4%), clindamycin (44.4%), and tetracycline (41.7%), with 47.2% MDR isolates. *E. coli* showed resistance to amoxicillin/clavulanic acid (88.9%), ampicillin (55.6%), and cefotaxime (55.6%), with 66.6% MDR. *S. aureus* remains the leading cause of subclinical mastitis in Western Romania. The high MDR rates highlight the need for targeted antimicrobial stewardship, culture-based therapy, and continuous AMR monitoring under the “One Health” framework.

## 1. Introduction

Bovine mastitis remains one of the most economically detrimental diseases of dairy cattle, causing significant reductions in milk yield and quality, increased treatment costs, and premature culling of affected animals [1,2,3]. Although both clinical and subclinical forms are encountered in dairy herds, subclinical mastitis is particularly challenging due to its asymptomatic nature, leading to undetected infections that persist within the herd and contribute to chronic productivity losses [4,5]. Effective control of subclinical mastitis, therefore, relies on timely identification of the causative pathogens and their antimicrobial susceptibility patterns [6,7].

A wide range of bacterial species have been implicated in bovine mastitis, but the majority of intramammary infections are caused by a limited number of pathogens, particularly *Staphylococcus aureus*, *Escherichia coli*, *Klebsiella pneumoniae* subsp. *pneumoniae*, and *Streptococcus uberis* [8,9]. These species represent both contagious and environmental agents, reflecting complex infection dynamics influenced by milking hygiene, housing conditions, and farm management practices. *S. aureus* and *S. uberis* are often associated with persistent, difficult-to-treat infections due to their ability to evade host defenses and form biofilms [10,11], while *E. coli* and *K. pneumoniae* are typically environmental opportunists linked to poor hygiene or contaminated bedding materials [12]. Understanding the distribution of these pathogens is therefore essential for developing targeted preventive and therapeutic strategies.

In recent years, the growing concern over antimicrobial resistance (AMR) among mastitis pathogens has added a critical dimension to bovine mastitis control. The frequent and sometimes empirical use of antimicrobials in dairy production has accelerated the selection of resistant strains, including multidrug-resistant (MDR) *S. aureus* and *E. coli* [13,14]. Such strains compromise treatment efficacy, prolong infection persistence, and may also represent a potential public health threat through the dissemination of resistance determinants via the food chain or environment [15]. Continuous regional monitoring of AMR in major mastitis pathogens is thus vital for informing evidence-based antimicrobial stewardship and aligning veterinary practices with the “One Health” perspective [1,16,17].

Although several studies have reported the occurrence of mastitis and AMR in Western Europe, data from Eastern European countries, including Romania, remain comparatively scarce and fragmented, particularly for subclinical cases [18,19]. In Romania, investigations into bovine mastitis etiology and AMR have increased in recent years, yet available data remain regionally fragmented and are often focused on clinical cases. Studies conducted in Western and Central Romania have consistently identified *S. aureus* as the predominant mastitis-associated pathogen, followed by variable contributions of environmental bacteria such as *E. coli*, *K. pneumoniae*, and *S. uberis* [19,20,21]. Western Romania, characterized by intensive dairy production systems, therefore represents a particularly relevant setting for investigating the etiological diversity and AMR profiles of mastitis-associated bacteria under real-world herd management conditions [19,20]. Recent Romanian and international investigations indicate that the molecular epidemiology of subclinical mastitis is shifting toward increased diversity of *S. aureus* lineages and environmental Gram-negative pathogens, frequently harboring mobile genetic elements conferring MDR [21,22,23,24]. In parallel, European AMR surveillance programs and Romanian reports have documented increasing resistance to β-lactams, tetracyclines, and macrolides among bovine mastitis isolates, underscoring the urgent need for updated, region-specific data to guide evidence-based mastitis control, antimicrobial stewardship [25], and national AMR monitoring strategies [21,25].

The present study aimed to characterize the etiological profile of subclinical mastitis in dairy cows in Western Romania by using ChromID^®^ CPS^®^ Elite Agar for selective differentiation and identification of major mastitis pathogens *E. coli*, *K. pneumoniae* subsp. *pneumoniae*, *S. aureus* subsp. *aureus*, and *S. uberis*. In addition, the antimicrobial susceptibility of *S. aureus* and *E. coli* isolates was evaluated using the automated Vitek^®^2 system to assess resistance patterns of key veterinary and zoonotic concerns. The findings provide updated insights into the bacterial spectrum and AMR trends of subclinical mastitis in the region, supporting improved diagnostic, therapeutic, and preventive strategies for sustainable dairy herd management.

## 2. Materials and Methods

### 2.1. Study Design and Population

The study was conducted over two consecutive years (2021–2022) on three dairy farms in Western Romania: Farm A (Bihor County; 120 cows), Farm B (Timiș County; 110 cows), and Farm C (Bihor County; 80 cows). A total of 117 lactating cows with subclinical mastitis were enrolled: 63 from Farm A, 39 from Farm B, and 15 from Farm C. The herds consisted of Holstein, Red Holstein, and Romanian Spotted Cattle breeds. All cows were housed in a free-stall system, which allowed free movement and continuous access to feed and water. Milking was performed automatically in dedicated milking parlors in each farm. A uniform mixed-feeding regimen, adjusted to the average milk yield of each group, was implemented across all three farms and administered three times daily.

Sampling was not performed using proportional random selection at the farm level. Instead, enrolment was based on the identification of cows meeting the inclusion criteria for subclinical mastitis during routine herd-health visits. As a result, the number of sampled cows per farm primarily reflected the observed occurrence of subclinical mastitis at the time of herd-health visits, rather than herd size or proportional representation.

### 2.2. Sample Size Determination

Cochran’s formula [26] was used solely to estimate a theoretical minimum sample size for prevalence-based studies, assuming random sampling, in order to provide a contextual benchmark for the adequacy of the final number of enrolled cows. Using a previously reported prevalence of *Staphylococcus* spp. in the region (*p* = 0.431) [19], a 95% confidence level (Z = 1.96), and a 5% margin of error (e = 0.05), the calculated sample size was n_0_ ≈ 377. After applying the finite population correction for the total number of eligible cows on the three farms (N = 117), the adjusted minimum sample size was approximately 90 cows. However, because the present study employed a convenience sampling approach based on the identification of cows with subclinical mastitis during routine herd-health visits, Cochran’s formula was not used to guide sampling or cow selection. Instead, all eligible cows meeting the inclusion criteria during the study period (n = 117) were enrolled. The calculation is therefore presented only to demonstrate that the final sample size exceeded the minimum number theoretically required for prevalence estimation.

### 2.3. Sample Collection and Diagnosis of Subclinical Mastitis

The study followed a cross-sectional design at the individual cow level. Lactating cows were eligible for inclusion if they showed no clinical signs of mastitis but tested positive with the Keno™ TEST (Cid Lines^®^, Ieper, Belgium) on fore-milk samples collected before routine milking. Likewise, each lactating cow was eligible for inclusion only once during the entire study period (2021–2022), and repeated sampling of the same animal was not performed. Screening for subclinical mastitis using the Keno™ TEST was conducted during scheduled herd-health visits rather than before every milking. Individual animal identification records were used to ensure that cows testing positive at different time points were not re-enrolled. Consequently, each positive cow contributed a single composite milk sample to the study.

The screening was performed on-farm by the veterinarians responsible for herd-health management, who also interpreted the test results according to the manufacturer’s instructions. For each cow, individual fore-milk samples were aseptically collected from all four quarters and pooled into a single sterile tube, resulting in one composite milk sample per animal. All bacteriological analyses and antimicrobial susceptibility testing were therefore performed on pooled cow-level samples, and results are expressed per cow (n = 117). Samples were stored at 4 °C and transported within 4 h to the Microbiological Risk Assessment Laboratory of the Faculty of Veterinary Medicine, Timișoara, where microbiological analyses were subsequently performed. Briefly, for Keno™ TEST screening, a few drops of fore-milk from each quarter were mixed with an equal volume of reagent on a clean paddle, and the formation of a gel-like viscosity within 10–15 s was interpreted as a positive reaction.

It should be noted that field screening tests, such as the Keno™ TEST, have a lower detection sensitivity compared with laboratory-based somatic cell count determination and may not identify low-grade subclinical infections with somatic cell counts close to physiological thresholds.

### 2.4. Etiological Identification of Bacterial Agents

Milk samples from positive cows were further cultured for etiologic identification on ChromID^®^ CPS^®^ Elite Agar (*Biomérieux*, *Pioneering Diagnostics*), which selectively differentiates major mastitis-associated Gram-negative and Gram-positive pathogens. After incubation at 37 °C for 24–48 h under aerobic conditions, colonies were examined according to the manufacturer’s color–morphology chart to identify *E. coli*, *K. pneumoniae* subsp. *pneumoniae*, *S. aureus* subsp. *aureus*, and *S. uberis*. A pooled sample was considered culture-positive when at least one colony-forming unit (CFU) of a potential mastitis pathogen was detected after incubation on ChromID^®^ CPS^®^ Elite Agar, indicating the presence of viable bacteria in the composite milk sample. Following preliminary differentiation on ChromID^®^ CPS^®^ Elite Agar (bioMérieux, Marcy l’Etoile, France), all isolates were identified to the species level using the Vitek 2 Compact automated system (bioMérieux, Marcy l’Etoile, France) with the Vitek 2^®^ ID-GN and Vitek2^®^ ID-GP cards, based on their biochemical profiles. From each positive culture plate, up to two morphologically distinct colonies were selected for further identification to ensure representation of all observable colony types. A sample was considered a mixed infection when two or more distinct bacterial morphotypes, confirmed as different species, were isolated from the same pooled milk sample.

Although molecular confirmation methods such as species-specific PCR or 16S rRNA gene sequencing can further increase diagnostic resolution, they were not applied in the present study due to its applied, surveillance-focused design and resource constraints. However, the combined use of chromogenic media and automated biochemical identification is a widely accepted and validated approach for routine identification of major mastitis-associated pathogens.

### 2.5. Antimicrobial Susceptibility Testing of the Isolates

Among the bacterial species isolated, antimicrobial susceptibility testing was deliberately restricted to *S. aureus* subsp. *aureus* and *E. coli.* These species are recognized as the principal etiological agents of bovine mastitis and are of significant veterinary and zoonotic importance [27,28,29,30]. Furthermore, standardized veterinary interpretive criteria (CLSI) and validated automated AST procedures using the Vitek^®^2 system are well established for these organisms [27,28,29,30,31]. All isolates of *S. aureus* (n = 36) and *E. coli* (n = 9) recovered during the study period were included in AST. In contrast, AST was not performed for *Klebsiella pneumoniae* subsp. *pneumoniae* and *Streptococcus uberis* because of their low isolation frequency and the limited availability of harmonized veterinary-specific interpretive criteria for automated susceptibility testing, which could compromise the reliability and interpretability of resistance estimates.

Antimicrobial susceptibility testing (AST) was performed on 36 *S. aureus* and 9 *E. coli* isolates recovered from subclinical mastitis cow milk using the fully automated Vitek2 system (bioMérieux, Marcy-l’Étoile, France). For *S. aureus*, the AST-P592 diagnostic card was used to assess susceptibility to 17 antimicrobial compounds spanning multiple classes, namely aminoglycosides (gentamicin [GEN; 0.5–16 µg/mL]); β-lactams (benzylpenicillin [PCG; 0.03–0.5 µg/mL], oxacillin [OXA; 0.25–4 µg/mL], and imipenem [IPM; 1–16 µg/mL]); fluoroquinolones (ciprofloxacin [CIP; 0.5–8 µg/mL] and moxifloxacin [MXF; 0.25–8 µg/mL]); glycopeptides (teicoplanin [TEC; 0.5–32 µg/mL] and vancomycin [VAN; 0.5–32 µg/mL]); lincomycins (clindamycin [CLY; 0.25–8 µg/mL]); macrolides (erythromycin [ERY; 0.25–8 µg/mL]); oxazolidinones (linezolid [LZD; 0.5–8 µg/mL]); phosphonic acid derivatives (fosfomycin [FOF; 8–128 µg/mL]); rifamycins (rifampicin [RIF; 0.5–32 µg/mL]); steroids (fusidic acid [FA; 0.5–32 µg/mL]); sulfonamides (trimethoprim–sulfamethoxazole [SXT; 10–320 µg/mL]); and tetracyclines (tetracycline [TET; 1–16 µg/mL] and tigecycline [TGC; 0.12–2 µg/mL]).

For *E. coli*, the AST-N204 diagnostic card was employed, covering a wide spectrum of antimicrobial agents, including aminoglycosides (amikacin [AMK; 2–64 µg/mL] and gentamicin [GEN; 1–16 µg/mL]); β-lactams (amoxicillin/clavulanic acid [AMC; 2/1–32/16 µg/mL], ampicillin [AMP; 2–32 µg/mL]), cephalosporins (cefepime [CPM; 1–64 µg/mL], cefotaxime [CTX; 1–64 µg/mL], ceftazidime [CAZ; 1–64 µg/mL], carbapenems (ertapenem [ERT; 0.5–8 µg/mL], imipenem [IPM; 0.25–16 µg/mL], and meropenem [MEM; 0.25–16 µg/mL]); fluoroquinolones (ciprofloxacin [CIP; 0.25–4 µg/mL] and norfloxacin [NOR; 0.5–16 µg/mL]); nitrofurans (nitrofurantoin [NIF; 16–512 µg/mL]); phosphonic acid derivatives (fosfomycin [FOF; 16–256 µg/mL]); and sulfonamides (trimethoprim–sulfamethoxazole [SXT; 20–320 µg/mL]).

For both species, the Vitek2 system automatically calculates minimum inhibitory concentrations (MICs) and classifies each isolate as susceptible, intermediate, or resistant using established thresholds.

MDR was characterized as the acquisition of resistance to at least one agent in three or more distinct antimicrobial categories, in accordance with the definition proposed by Magiorakos et al. [32]. Antimicrobial susceptibility was interpreted using the clinical breakpoints specified by the Clinical and Laboratory Standards Institute (CLSI) [33]. To ensure the reliability of the susceptibility assays, *S. aureus* ATCC^®^ 29213™ and *E. coli* ATCC^®^ 25922™ were employed as internal quality control reference strains.

### 2.6. Statistical Analysis

Contingency analyses were performed in SPSS version 23 (IBM Corp., Armonk, NY, USA). For comparisons of categorical variables among farms, we used χ^2^ tests when all expected counts exceeded 5 and Fisher’s exact test otherwise. *p* < 0.05 was considered statistically significant. Between-farm comparisons were therefore interpreted descriptively and inferentially within the context of an observational, non-random sampling design.

## 3. Results

Out of the 117 milk samples collected from cows diagnosed with subclinical mastitis, 51 (43.6%; 95% Confidence Interval: 34.9–52.6) yielded positive bacterial growth following culture on ChromID^®^ CPS^®^ Elite Agar (Table 1). The highest proportion of culture-positive samples was recorded in Farm C (60.0%), followed by Farm B (43.6%) and Farm A (39.7%), though the differences were not statistically significant (χ^2^ = 2.18, *p* > 0.05).

Among the isolates, *S. aureus* subsp. *aureus* was the predominant pathogen, being identified in 33 samples (28.2% of all cows; 95% CI: 20.8–36.9), with the highest frequency observed in Farm A (16/63; 25.4%) and Farm B (12/39; 30.8%). *E. coli* was isolated from 5 cows (4.3%; 95% CI: 1.8–9.6), *K. pneumoniae* subsp. *pneumoniae* from 3 cows (2.6%; 95% CI: 0.9–7.3), and *S. uberis* from 3 cows (2.3%; 95% CI: 0.9–7.3).

Mixed infections were detected in a small proportion of samples (n = 7; 6.0%), with the most frequent associations being *S. aureus* + *K. pneumoniae* subsp. *pneumoniae* (n = 2) and *E. coli* + *S. uberis* (n = 2). Less common combinations included *S. aureus* + *E. coli*, *E. coli* + *K. pneumoniae* subsp. *pneumoniae*, and *K. pneumoniae* subsp. *pneumoniae* + *S. uberis* (each n = 1). The occurrence of mixed infections did not differ significantly among the investigated farms (*p* > 0.05).

On ChromID^®^ CPS^®^ Elite Agar, the isolated bacteria displayed distinct colony morphologies, facilitating preliminary differentiation: *S. aureus* subsp. *aureus* formed yellowish-white colonies with variable shades, *K. pneumoniae* subsp. *pneumoniae* exhibited light bluish-gray colonies, *S. uberis* developed dark blue colonies, whereas *E. coli* produced red to burgundy-red colonies.

Overall, *S. aureus* subsp. *aureus* was confirmed as the leading etiological agent of subclinical mastitis in all three farms, followed by *E. coli*, *K. pneumoniae*, and *S. uberis*. The moderate rate of mixed infections suggests possible polymicrobial interactions within the mammary gland microenvironment, though without significant inter-farm variation.

Antimicrobial susceptibility testing (AST) was successfully performed on 36 *S. aureus* and 9 *E. coli* isolates obtained from bovine subclinical mastitis milk samples using the automated Vitek^®^2 system. Quality control strains *S. aureus* ATCC 29213™ and *E. coli* ATCC 25922™ were within QC ranges during testing. The obtained resistance profiles revealed notable inter- and intra-species variability, with a substantial proportion of isolates expressing MDR phenotypes.

Among the 36 *S. aureus* isolates tested with the AST-P592 diagnostic card, the highest resistance frequencies were observed against FOF (58.3%; 21/36), PCGn (44.4%; 16/36), CLI (44.4%; 16/36), TET (41.7%; 15/36), and ERY (38.9%; 14/36). Moderate resistance was recorded for FA (16.7%; 6/36), OXA (13.9%; 5/36), CIP (11.1%; 4/36), RIF (11.1%; 4/36), GEN (5.6%; 2/36), and TEC (5.6%; 2/36). All isolates remained susceptible to IPM, MXF, VAN, LZD, SXT, and TCG. Overall, seven isolates (19.4%) were fully susceptible to all tested antimicrobials, whereas 17 (47.2%; 95% C.I.: 32.0–63.0) met the criteria for MDR, exhibiting resistance to at least one compound in three or more antimicrobial classes. The remaining strains displayed resistance to one or two antimicrobial categories.

Detailed examination of resistance combinations revealed a diverse range of MDR phenotypes (Table 2). The most complex MDR pattern included simultaneous resistance to β-lactams, aminoglycosides, lincomycins, macrolides, phosphonic acid derivatives, and tetracyclines. Other common profiles combined β-lactams with macrolides, phosphonic acid derivatives, and tetracyclines, or the co-occurrence of fluoroquinolone and rifamycin resistance. These heterogeneous patterns suggest a mosaic structure of resistance determinants among the mastitis-associated *S. aureus* isolates.

MDR was detected in six isolates (66.6%), each showing resistance to at least three antimicrobial classes. Three isolates expressed combined resistance to β-lactams, cephalosporins, phosphonic acid derivatives, and sulfonamides, while two others exhibited resistance to β-lactams, cephalosporins, and fluoroquinolones. One isolate displayed a simpler MDR profile involving β-lactams, phosphonic acid derivatives, and sulfonamides. One strain remained fully susceptible to all tested agents.

The MDR patterns identified among *S. aureus* and *E. coli* isolates are summarized in Table 2. In *S. aureus*, the most frequent MDR combinations involved β-lactams, macrolides, and phosphonic acid derivatives, often accompanied by resistance to tetracyclines or lincomycins. In contrast, *E. coli* isolates predominantly exhibited co-resistance to β-lactams, cephalosporins, and either sulfonamides or fosfomycin, which may reflect selective pressure from β-lactam use. However, antimicrobial usage data were not collected, so causality cannot be inferred.

These findings highlight the circulation of diverse MDR phenotypes among bovine mastitis pathogens, underlining the need for continued antimicrobial surveillance and prudent use of antimicrobials in veterinary practice.

## 4. Discussion

The present study provides updated regional data on the etiological agents and AMR profiles associated with subclinical mastitis in dairy cows from Western Romania. Our finding that *S. aureus* subsp. *aureus* was the most frequently isolated pathogen is consistent with prior reports that identify *S. aureus* as a dominant cause of subclinical intramammary infections in intensively managed dairy systems [19,20]. The predominance of *S. aureus* in subclinical presentations likely reflects its well-described ability to persist in the udder through biofilm formation and intracellular survival, mechanisms which facilitate chronic, often asymptomatic infection [11,34]. Persistent *S. aureus* carriage is an important driver of herd-level transmission and long-term production losses in subclinical disease [19].

Comparison with existing Romanian literature indicates that our results are consistent with national epidemiological patterns while providing updated insight into subclinical mastitis. Several Romanian studies have reported *S. aureus* prevalence ranging between 25% and 45% among mastitis isolates, confirming its long-standing dominance in dairy herds across different production systems [19,20,21]. The prevalence observed in the present study (28.2%) falls within this range and further supports the role of *S. aureus* as a key pathogen in subclinical intramammary infections. Lower isolation frequencies of environmental pathogens such as *E. coli*, *K. pneumoniae*, and *S. uberis* are also consistent with Romanian findings, where these bacteria are typically associated with hygiene-related risk factors rather than persistent contagious transmission [19,21].

Less frequent recovery of *E. coli*, *K. pneumoniae* subsp. *pneumoniae*, and *S. uberis* follows typical patterns for subclinical mastitis, where contagious pathogens such as *S. aureus* dominate while many Gram-negative organisms remain comparatively uncommon unless environmental risk factors are present [9,29]. Environmental coliforms like *E. coli* and *Klebsiella* typically proliferate where bedding and hygiene are suboptimal. Thus, their lower prevalence in our sample may suggest generally adequate environmental management in the studied herds [29]. Nonetheless, the presence of these opportunistic environmental pathogens, even at low frequencies, highlights the need to maintain environmental control measures alongside contagious-pathogen control programs [6].

Mixed infections were identified in a small fraction of samples (6.0%), a rate similar to those reported in other herd-level surveys of subclinical intramammary infections [9]. Polymicrobial intramammary infections can complicate host responses and therapeutic outcomes because of potential inter-species interactions (e.g., co-aggregation in biofilms) and horizontal transfer of mobile genetic elements. However, the clinical significance of low-frequency mixed infections in subclinical contexts remains to be fully elucidated [11].

Because sampling was conducted continuously throughout all months of 2021–2022, potential seasonal effects on pathogen distribution or prevalence were minimized.

The antimicrobial susceptibility profiles obtained with the automated Vitek^®^2 system revealed important resistance trends. Among the *S. aureus* isolates, we observed high resistance frequencies to FOF, PCG, CLY, TET, and ERY. These results align with recent surveillance that reports persistent β-lactam and macrolide/lincosamide resistance among bovine *S. aureus* isolates [13]. The high proportion of *S. aureus* isolates that fulfilled conventional MDR criteria (47.2%) is notable and raises concerns about limited therapeutic options at the herd level; similar MDR frequencies have been flagged in regional studies and emphasize the need for targeted stewardship [13,15].

From an antimicrobial resistance perspective, our findings align closely with recent Romanian studies reporting high resistance rates of mastitis-associated *S. aureus* to penicillin, tetracycline, and macrolides, as well as the presence of MDR phenotypes in dairy herds [19,20]. Huțu et al. [20] demonstrated widespread resistance and the circulation of resistance determinants among *S. aureus* isolates from Western Romanian farms, while Neculai-Valeanu et al. [35] emphasized the broader implications of antimicrobial resistance dissemination from dairy systems to public health. The MDR prevalence observed in our study, particularly among subclinical isolates, suggests that resistance may persist or increase in the absence of targeted diagnostic-guided interventions.

FOF resistance observed in a sizeable fraction of *S. aureus* isolates merits specific attention because fosfomycin has been revisited as a treatment option in some veterinary contexts and because resistance determinants may be co-localized with other AMR genes on mobile elements [13]. Mechanisms such as plasmid-borne *fos* genes and co-selection by other antimicrobials are plausible and warrant molecular investigation [21].

For *E. coli*, the most frequent resistances involved AMC, AMP, and extended-spectrum cephalosporins (e.g., CTX). This pattern is compatible with widespread β-lactamase production among bovine *E. coli* isolates as documented in both national and international surveillance reports [16,18]. The detection of co-resistance to sulfonamides and fosfomycin further suggests accumulation of multiple mobile resistance determinants in certain strains, a situation that increases the likelihood of MDR phenotypes [15,18]. Notably, all *E. coli* isolates in our sample remained susceptible to aminoglycoside and carbapenem agents that are rarely used in food-producing animals, indicating that restricted veterinary usage practices may help preserve susceptibility to these classes [27].

The observed MDR prevalence in *E. coli* (66.6%) is higher than some European averages reported in the literature [17], suggesting either local selection pressures or small sample effects, given the limited number of *E. coli* isolates tested [17,18]. This finding underscores the importance of culture-guided therapy rather than empirical broad-spectrum treatment, and of integrating antimicrobial stewardship measures at the farm level [6].

Our findings reinforce that *S. aureus* continues to be a major cause of subclinical mastitis in Western Romania and that MDR phenotypes among both *S. aureus* and *E. coli* are present in milk from apparently healthy cows [19,20]. Because raw milk can serve as a vehicle for AMR determinants, these results have implications beyond bovine health. The potential for environmental dissemination and zoonotic transfer of resistance genes aligns with concerns raised in European surveillance reports and global One Health assessments [17,30]. Consequently, regional surveillance data like ours are essential for informing national mitigation strategies and harmonizing veterinary antimicrobial policies with EU recommendations [17,18].

On a practical level, the data support the use of herd-level control measures that combine contagious-pathogen management (e.g., identification and management of persistent *S. aureus* shedders, post-milking teat disinfection) with environmental hygiene practices to reduce opportunistic infections [6,19]. From an antimicrobial stewardship perspective, adoption of culture-based therapy, selective dry-cow therapy, and farm-specific treatment protocols could reduce unnecessary antimicrobial exposure that drives resistance [6,18]. Although the present study focuses on bacterial pathogens associated with bovine subclinical mastitis, recent investigations have emphasized the potential of alternative antimicrobial and antibiofilm strategies to limit persistent bacterial populations in complex biological systems [35,36]. Such findings reinforce the broader concept that enhancing antimicrobial efficacy, whether in food matrices or biofilm-forming environments, may also improve therapeutic outcomes in chronic intramammary infections.

At the national level, the detection of MDR bacteria in milk from cows with subclinical mastitis reinforces concerns raised by recent Romanian and European studies regarding the silent spread of antimicrobial resistance within food-producing animal populations [19,20,35]. Subclinical mastitis represents a hidden but epidemiologically significant reservoir of resistant bacteria, with potential implications for herd productivity, environmental contamination, and public health. Our findings support calls for strengthened national surveillance of mastitis pathogens and AMR, integration of digital and diagnostic tools in herd health management, and alignment of Romanian dairy practices with EU One Health antimicrobial stewardship frameworks.

Future work should include molecular characterization (resistance genes, plasmid replicons, and virulence factors) and strain typing to determine clonal relationships and track potential zoonotic lineages [16,20]. Longitudinal monitoring at the herd level would also clarify the temporal dynamics of AMR and the effect of management interventions on resistance prevalence [15].

Several limitations should be acknowledged. Firstly, although the sample included all eligible subclinical cases detected on the three study farms, the geographic and herd coverage is limited to Western Romania and may not represent national patterns. Secondly, antimicrobial susceptibility testing was restricted to *S. aureus* and *E. coli*, so AMR data are lacking for *K. pneumoniae* and *S. uberis* from our isolates [27]. Third, the study did not include molecular analyses of resistance determinants or strain typing. Therefore, conclusions about the genetic basis for observed resistance phenotypes are speculative and should be confirmed in follow-up genomic studies [16,20]. While the combined use of ChromID^®^ CPS^®^ Elite Agar and Vitek^®^2 biochemical profiling provides reliable identification for common mastitis pathogens, future investigations should incorporate molecular techniques such as species-specific PCR or sequencing to further strengthen taxonomic accuracy and enable strain-level characterization. Also, because the study targeted only major mastitis-associated pathogens, infections caused by less common bacterial species may not have been detected, potentially leading to an underestimation of overall mastitis prevalence. Fourth, an important limitation of the present study relates to the use of a field-based screening test for the enrollment of cows with subclinical mastitis. Tests such as the Keno™ TEST are known to reliably detect inflammatory responses at somatic cell counts typically exceeding 300–500 × 10^3^ cells/mL, whereas infections associated with lower cell counts may remain undetected. Consequently, some cows harboring low-grade intramammary infections may not have been enrolled, while physiological increases in somatic cell counts related to stage of lactation cannot be completely excluded. However, as the study aimed to characterize the etiological profile and antimicrobial resistance patterns of field-detectable subclinical mastitis cases rather than to determine true disease prevalence, this limitation is unlikely to have influenced the main conclusions. Fifth, detailed herd-level antimicrobial usage histories and management variables were not systematically collected, limiting the ability to statistically link specific practices to AMR patterns [6]. Finally, the relatively small numbers of some species (e.g., *E. coli* n = 9) increase uncertainty around prevalence estimates and MDR proportions. Therefore, the observed resistance proportions should be interpreted with caution, as they may not fully represent the wider bacterial population or the regional resistance situation. Larger, multi-center studies would provide more robust estimates [17]. Despite these constraints, the findings provide valuable preliminary insight into the antimicrobial resistance characteristics of mastitis-associated bacteria in this region.

## 5. Conclusions

This study highlights *S. aureus* as the predominant etiological agent of subclinical mastitis in dairy cows in Western Romania, followed by *E. coli*, *K. pneumoniae*, and *S. uberis*. The identification of high rates of antimicrobial resistance, particularly MDR among both *S. aureus* and *E. coli* isolates, underscores the ongoing challenge of AMR in dairy production. Resistance to β-lactams, macrolides, tetracyclines, and fosfomycin reflects long-term selective pressures associated with routine antimicrobial usage in mastitis control. These findings emphasize the urgent need for region-specific antimicrobial stewardship programs, culture-based therapeutic decision-making, and continuous AMR monitoring as integral components of sustainable mastitis management. The data generated contribute valuable baseline information to national and European AMR surveillance efforts and reinforce the importance of coordinated “One Health” approaches to safeguard both animal and public health.

## Figures and Tables

**Table 1:** Distribution of bacterial pathogens isolated from milk samples of cows with subclinical mastitis (n = 117) according to the investigated farms.

Bacterial Species/Combination	Farm A (n = 63)	Farm B (n = 39)	Farm C (n = 15)	Total Positive Samples (n)	Proportion of All Cows (%)
*S. aureus* subsp. *aureus*	16	12	5	33	28.2
*E. coli*	2	2	1	5	4.3
*K. pneumoniae* subsp. *pneumoniae*	2	-	1	3	2.3
*S. uberis*	1	1	1	3	2.3
Mixed infections					
*S. aureus* + *K. pneumoniae*	2	-	-	2	1.7
*S. aureus* + *E. coli*	-	1	-	1	0.9
*E. coli* + *K. pneumoniae*	-	-	1	1	0.9
*E. coli* + *S. uberis*	2	-	-	2	1.7
*K. pneumoniae* + *S. uberis*	-	1	-	1	0.9
Total cows with positive bacterial growth (%)	25 (39.7)	17 (43.6)	9 (60.0%)	51 (43.6)	100%

**Table 2:** The combination of MDR phenotypes observed in the tested *S. aureus* and *E. coli* isolates.

Bacteria	No. of Classes with Resistance	Number of Isolates	Classes with Resistance	AMR Profile of the Isolated Strains
*S. aureus*	6	2	β – lactams + aminoglycosides + lincomycins + macrolides + phosphonic acid derivatives + tetracyclines	PCG + OXA + GEN + CLY + ERY + FOF + TET
	5	1	β − lactams + lincomycins + macrolides + phosphonic acid derivatives + tetracyclines	PCG + CLY + ERY + FOF + TET
	5	3	β − lactams + fluoroquinolones + macrolides + phosphonic acid derivatives + steroids	PCG + CIP + ERY + FOF + FA
	5	1	fluoroquinolones + macrolides + phosphonic acid derivatives + rifamycins + tetracyclines	CIP + ERY + FOF + RIF + TET
	4	2	β − lactams + lincomycins + macrolides + tetracyclines	PCG + OXA + CLY + ERY + TET
	4	2	β − lactams + glycopeptides + phosphonic acid derivatives + tetracyclines	PCG + TEC + FOF + TET
	4	1	β − lactams + lincomycins + macrolides + tetracyclines	PCG + CLY + ERY + TET
	4	1	β − lactams + lincomycins + phosphonic acid derivatives + steroids	PCG + OXA + CLY + FOF + FA
	4	4	lincomycins + macrolides + phosphonic acid derivatives + tetracyclines	CLY + ERY + FOF + TET
*E. coli*	4	3	β − lactams + cephalosporins + phosphonic acid derivatives + sulfonamides	AMC + AMP + CPM + CTX + CAZ + FOF + STX
	3	2	β − lactams + cephalosporins + fluoroquinolones	AMC + CTX + NOR
	3	1	β − lactams + phosphonic acid derivatives + sulfonamides	AMC + AMP + FOF + STX

Legend: The diagnostic card demonstrated frequent resistance to β-lactams and extended-spectrum cephalosporins. The highest resistance rate was observed for AMC (88.9%; 8/9), followed by AMP (55.6%; 5/9), CTX (55.6%; 5/9), FOF (55.6%; 5/9), and STX (44.4%; 4/9). Moderate resistance occurred to NOR (33.3%; 3/9), CAZ (33.3%; 3/9), and CPM (33.3%; 3/9). All isolates were susceptible to AMK, GEN, ERT, IPM, MEM, CIP, and NIF.

## Data Availability

All data generated or analyzed during this study are included in the submitted version of the manuscript.
